# Predicting the Effect of Temperature Changes on *Phlebotomus papatasi* Activity, as the Main Vector of Zoonotic Cutaneous Leishmaniasis in Iran

**DOI:** 10.1155/tbed/9518371

**Published:** 2025-01-15

**Authors:** Faramarz Bozorg-Omid, Fahimeh Youssefi, Gholamreza Hassanpour, Abbas Rahimi Foroushani, Mohammad Rahimi, Mohammad Reza Shirzadi, Reza Jafari, Ahmad Ali Hanafi-Bojd

**Affiliations:** ^1^Department of Vector Biology and Control of Diseases, School of Public Health, Tehran University of Medical Sciences, Tehran, Iran; ^2^Institute of Artificial Intelligence, Shaoxing University, Shaoxing, Zhejiang Province Postal Code 312000, China; ^3^Department of Photogrammetry and Remote Sensing, Faculty of Geodesy and Geomatics Engineering, K. N. Toosi University of Technology, Tehran, Iran; ^4^Center for Research of Endemic Parasites of Iran (CREPI), Tehran University of Medical Sciences, Tehran, Iran; ^5^Department of Epidemiology and Biostatistics, School of Public Health, Tehran University of Medical Sciences, Tehran, Iran; ^6^Department of Combat Desertification, Faculty of Desert Studies, Semnan University, Semnan, Iran; ^7^Esfahan Research Center, School of Public Health, Tehran University of Medical Sciences, Esfahan, Iran; ^8^Zoonoses Research Center, Tehran University of Medical Sciences, Tehran, Iran

**Keywords:** climate change, cutaneous leishmaniasis, monthly patterns, *Phlebotomus papatasi*, sandfly activity

## Abstract

Cutaneous leishmaniasis (CL) represents a significant vector-borne disease in Iran. Our study examined the status of zoonotic CL (ZCL) in the country and forecasted the influence of global climate change on the monthly activity of *Phlebotomus papatasi*, the main vector of ZCL in the country. To predict the impact of climate change on the monthly activity of *Ph. papatasi*, we obtained the monthly average minimum and maximum temperatures for both the reference and future periods, using the MIROC6 model and two different shared socioeconomic pathway (SSP) scenarios. Based on our analysis, we found that *Ph. papatasi* can be active in Iran from March to November, although this may vary depending on the region. Our predictions suggest that the duration of *Ph. papatasi*'s activity may change following future changes in weather patterns. In different scenarios, the duration of the active season in various regions of the country extends by at least 1–2 months. This extension is likely more pronounced in the southern provinces. Additionally, our findings indicate a notable correlation between ZCL incidence, the presence of *Ph. papatasi*, and environmental factors in Ardestan, Esfahan Province. This study focuses on the impact of temperature on the activity and distribution of *Ph. papatasi* in Iran, which is a significant vector for transmitting ZCL. The study predicts that with future climate scenarios, especially SSP5-8.5, the activity of this vector will start earlier, last longer, and might even occur throughout the year by the 2050s, thereby increasing the risk of ZCL transmission. Although temperature plays a dominant role in shaping the activity of *Ph. papatasi*, its influence is not consistent across Iran. The variation in different regions emphasizes the importance of implementing targeted public health approaches to address the changing risks of ZCL transmission due to evolving climate conditions. However, it acknowledges that certain factors such as land use and humidity have not been taken into account and requests additional research in these areas. It also calls for enhanced environmental monitoring and public health interventions.

## 1. Introduction

Leishmaniasis is a worldwide vector-borne disease (VBD) that is transmitted to humans by the bite of infected female sand flies with *Leishmania* parasite species [1]. Although not all cases are reported to the World Health Organization (WHO) and cases may be manifold, it is estimated that 700,000–1 million new cases occur worldwide annually. More than 90 sand fly species are known as vectors, which can cause different clinical manifestations of leishmaniasis [2]. Cutaneous leishmaniasis (CL) is the most common form of leishmaniases, which is transmitted through the bite of phlebotomine sand flies. CL mainly affects regions in the Americas, the Mediterranean, the Middle East, and Central Asia, with 85% of cases in 2018 concentrated in 10 countries, including Afghanistan, Brazil, and Iran. Annually, an estimated 600,000 to 1 million new cases are reported worldwide [2]. In this region, Iran is one of the countries with a high annual incidence rate, where the incidence of CL has been reported to be 15.8 cases per 100,000 population and the burden of CL was between 1.18 and 5.7 disability-adjusted life years (DALYs) per 100,000 population in 2019 [2, 3]. Currently, CL is the most significant VBD in Iran, with epidemiological estimates attributing over 80% of cases to *Leishmania major* [4] (also known as zoonotic CL [ZCL] or rural CL). To date, 18 out of 31 provinces in Iran are foci for ZCL, while 8 provinces are affected by anthroponotic CL (ACL) [5, 6]. Confirmed CL cases are typically diagnosed at the genus level; however, for research purposes, investigators may identify cases down to the species level when needed [4].

Studies have shown that ZCL is influenced by a complex interplay of biological, socioeconomic, and environmental factors. General risk factors include the presence of the sandfly vector, *Phlebotomus* spp., the animal reservoir (primarily rodents), and human behavior that brings people into contact with these vectors. Socioeconomic drivers, including poverty and illiteracy, exacerbate the spread of ZCL by increasing human–vector interactions and reducing effective disease control [7]. Furthermore, topographical factors, such as elevation, latitude, longitude, and land cover, also shape the distribution of *Ph. papatasi*. For instance, concerning the latitude, *Ph. papatasi* distribution, the high number is localized between 27° and 35° [8]. Nevertheless, environmental and climatic factors such as temperature have been shown to have a more substantial impact on ZCL transmission cycles. For instance, climatic variables can directly affect vector population dynamics, survival, and breeding and indirectly influence human–vector contact by altering agricultural patterns and human settlements, and studies have also demonstrated that climate variability, particularly rising temperatures and erratic rainfall, has led to an expansion of ZCL into new regions. Thus, while human and socioeconomic factors play a significant role, environmental and climatic factors are critical in determining the spatial and temporal distribution of ZCL [9]. The pattern of ZCL observed in Iran indicates a monthly disease pattern, based on retrospective data from different years. Using these historical data, models were applied to predict that with changing environmental conditions, the prevalence of ZCL is expected to shift in the future [10–14]. ZCL is mostly reported in autumn, likely due to a 2–4 months' delay between when infected sandflies bite individuals in early summer and when symptoms of the disease appear [15].

It has been claimed that the monthly ZCL pattern is also influenced by the monthly activity of vectors [8]. *Phlebotomus papatasi* is known as the main vector of ZCL in Iran [16] and many other countries [17–20]. The temporal distribution of *Ph. papatasi* is affected by the monthly change in minimum and maximum air temperature [8]. The temperature thresholds vary by species, but in general, laboratory studies in Iran have shown that the survival of *Ph. papatasi* is in the temperature ranges of 17.63–35°C [21]. When moving from one region or climate to another, there are changes in the maximum and minimum temperature, which lead to a change in the spatial–temporal pattern of the *Ph. papatasi* [8]. Also, with the suitable temperature in different months of the year, the activity of *Ph. papatasi* reaches its peak. For instance, this species is active mostly from June to October in most regions of Iran [22].

In the future, Iran is probably one of the countries expected to experience warmer temperatures due to climate change, so it has been predicted that the mean temperature will possibly increase between 1.12 and 7.87°C in the country by 2100 [23]. It is predicted that climate change will affect the distribution of *Ph. papatasi* in Iran so that the suitable environment for this species will expand in some areas [24]. It is also known that climate change can affect the monthly activity of vectors throughout the year [25], which can lead to a change in the ZCL pattern in the regions vulnerable to climate change. It has been reported in some parts of the world that increased minimum temperature following global warming has created conditions suitable for vector activity and prevalence of ZCL [26]. Almost all the studies conducted in Iran examined the change in the activity pattern of *Ph. papatasi* only under current conditions [22, 27, 28]. Previous studies suggest that climate change cannot only impact the geographic range of *Ph. papatasi* in Iran but also likely influence its activity levels and the duration of its active month [24].

To study possible future impacts, the Intergovernmental Panel on Climate Change (IPCC) in its sixth report relies on different scenarios called shared socioeconomic pathways (SSPs) [29]. The different global circulation models (GCMs), that is, Coupled Model Intercomparison Project Phase 6 (CMIP6), are available that simulate future climate changes according to different scenarios. This is an international collaborative framework that coordinates climate model experiments across the world. It provides a wide variety of climate models designed to simulate the Earth's climate system under different scenarios. The models used in CMIP6 incorporate many different kinds of variables, including greenhouse gas concentrations, aerosols, and land use changes, to project climate conditions in the future. This approach ensures that the predictions are strong and reliable, accounting for uncertainties in future emissions and other factors that likely influence climate [30]. A Japanese modeling community has cooperatively developed a new ocean–atmosphere coupled model called the sixth version of the Model for Interdisciplinary Research on Climate (MIROC6). In other words, it is a specific model within the CMIP6 suite, because it is well-regarded for its high resolution and its ability to simulate detailed climate processes, such as monsoons and atmospheric circulation patterns, which are crucial for understanding regional climate dynamics in areas like Iran; it has motivated us to perform decadal predictions using the MIROC6 model [31].

This study aimed to model changes in the monthly activity pattern of *Ph. papatasi* species under different climate change scenarios. Such spatial–temporal analysis of *Ph. papatasi* is to understand the current epidemiological situation and the future burden of CL diseases. Moreover, we evaluated the predictions made under current conditions in one of the important foci of the ZCL in Iran. The results will provide a basis for its management and for the prevention and control of ZCL, as well as basic information for ecological studies of *Ph. papatasi* species.

## 2. Materials and Methods

### 2.1. Climatic Data

The climate data used in this study were sourced from WorldClim version v.2.1, a high-resolution global climate database that provides historical climate data as well as future climate projections. In the current study, climatic data (monthly average minimum and maximum temperature) of the reference period (1970–2000) and the future period (the 2030s and 2050s), considering the MIROC6 model and two different SSP scenarios (SSP1-2.6 and SSP5-8.5), were obtained from the WorldClim website at a spatial resolution of 30 s. Indeed, WorldClim is a database of spatially interpolated monthly climate data for global land areas at a very high spatial resolution, and it offers gridded weather and climate layers, derived from a combination of ground-based observations and satellite data. Ground-based observations come from a network of meteorological stations around the world that provide accurate measurements of climate variables such as temperature and precipitation. Satellite-based observations, including infrared assessments, contribute to the data, especially in areas with sparse ground-based stations. These satellites provide valuable information on surface temperatures and vegetation conditions, which helps in filling gaps in ground-based data. The data are interpolated using sophisticated algorithms to create continuous climate surfaces. In WorldClim v.2.1, a thin-plate smoothing spline interpolation method is employed. This technique uses the available climate data points (from both ground-based and satellite sources) to produce high-resolution climate surfaces that represent average monthly climate conditions [32]. These data can be used for mapping and spatial modeling (www.worldclim.org).

### 2.2. Projection of Monthly Activity of *Ph. papatasi* Under Climate Change

It found that *Ph. papatasi* is active between the 17.63 and 35°C temperature range [21]. So, ranges with a minimum temperature of 17.63°C and a maximum temperature of 35°C were displayed as suitable places for vector growth, and values higher and lower than the mentioned temperature range were considered as the unfavorable environment. The variance of the monthly average maximum and minimum temperature from the reference period was calculated using the ArcGIS v.10.5 software. The temperature changes between the current and future conditions were calculated, and the relevant graphs were drawn using Excel software. On the other hand, for the prediction of the potential activity and month lengths, future climatic variable data (monthly maximum and minimum temperature) were clipped using ArcGIS v.10.5 according to the border of Iran (as an analysis mask) and were obtained in binary based on optimal growth temperature of *Ph. papatasi*.

### 2.3. Evaluation of *Ph. papatasi* Activity Binary Model

**Figure 1:**
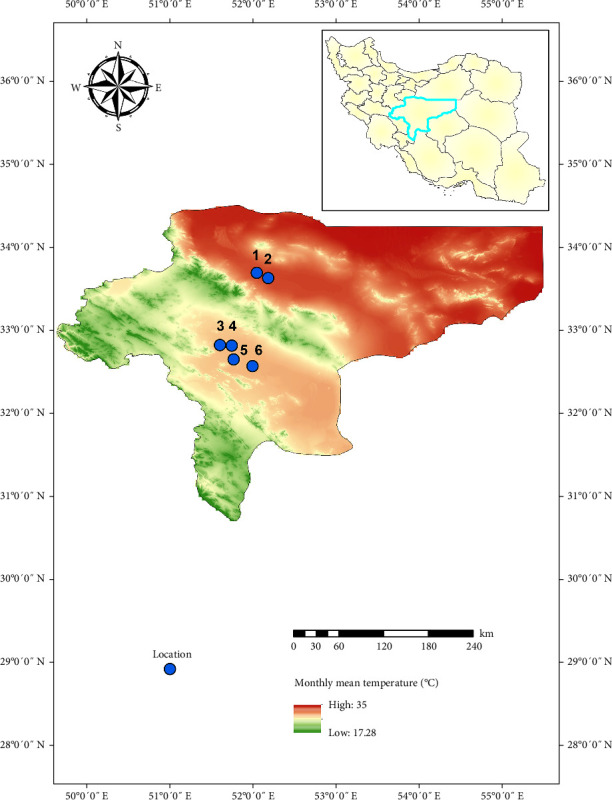
The location of the selected districts to collect *Phlebotomus papatasi* in Esfahan Province, July 2020. (1) Natanz (Moazi Abad, 52.051 N/33.6869011 E). (2) Ardestan (Nusrat Abad, 52.188 N/33.6231995 E). (3) Shahin Shahr (Chah Naji, 51.609 N/32.8172989 E). (4) Borkhar (Jurabi, 51.751 N/32.8087997 E). (5) Esfahan (Araghavanieh, 51.773 N/32.6423988 E). (6) Esfahan (Fassaran, 51.999 N/32.5620003 E). The map was generated using ArcGIS v10.5 (www.esri.com).

We evaluated the model in field conditions. We selected Esfahan Province because it is known as one of the most important foci of CL in Iran. Esfahan is located in the center of Iran, and it covers an area of ~107,000 km^2^ between 30°42′ and 34°30′ N latitude and 49°36′ and 55°32′ E longitude. In Esfahan Province, six districts were randomly selected. It should be noted that to avoid sampling bias in the evaluation of the modeling, we selected areas where no studies have been conducted regarding the presence of *Ph. papatasi* species until then (Figure 1). Specimens were collected from rodent burrows and near their breeding places during July when the temperature was optimal for the growth of the *Ph. papatasi* based on the reference period that obtained from the WorldClim website. Sampling was done once in each area with 40 sticky traps. In total, 240 sticky were installed in six districts. To specimens, the traps were collected and transported to the laboratory of Esfahan Health Research Station, Tehran University of Medical Sciences. The sand flies were identified based on morphometric characters.

### 2.4. Determination of CL Incidence

To determine the incidence of CL during 2011–2020 in different provinces of Iran, monthly statistics related to cases of the disease were received from the Zoonosis Department of the Centers for Disease Control and Prevention (CDC) of the Ministry of Health. On the other hand, data related to the population were received from the National Statistics Center of Iran (www.amar.org.ir). The incidence of the CL was calculated as cases per 100,000 population. To make the incidence distributions closer to the Gaussian distribution, the incidence of the CL was calculated using logarithm transformation in base 2 (log_2_) and log_2_ (incidence value + 1) command in R software. The annual maps of CL incidence during 2011–2020 were provided using ArcGIS v.10.5 and R software.

### 2.5. Relationships Between *Ph. papatasi* and Environmental Variables With ZCL Incidence

To determine the monthly activity of the vector and its role in disease occurrence, the monthly activity curve of *Ph. papatasi* was overlaid with the monthly occurrence of ZCL. To determine the monthly activity of *Ph. papatasi*, sampling is done every 15 days in a certain place using the sticky trap method. The number of sand flies caught in 30 traps is used as an indicator of vector abundance [33, 34]. Moreover, to investigate the statistical correlation between the ZCL incidence, with the number of *Ph. papatasi* and meteorological variables (monthly average temperature, monthly maximum, and minimum temperature), Poisson regression analysis was performed using SPSS v.19 software.

## 3. Results

### 3.1. The Impact of Temperature Changes on the Monthly Activity of *Ph. papatasi*

**Figure 2:**
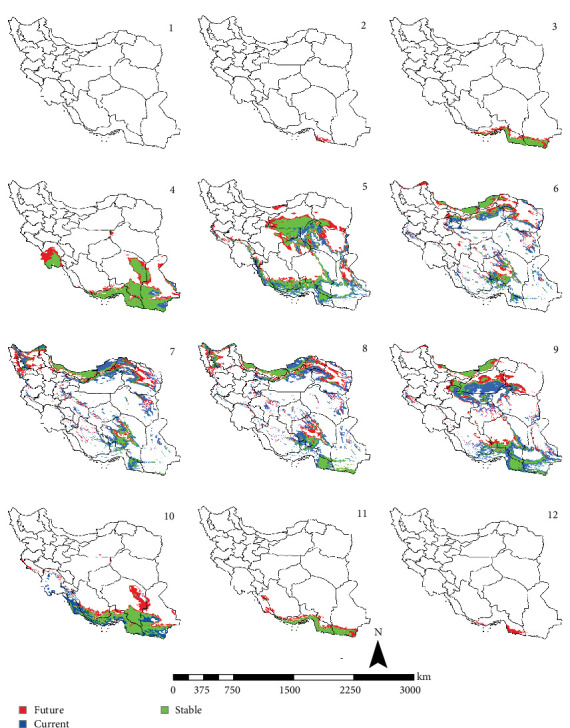
The projection of the activity of *Phlebotomus papatasi* under SSP1-2.6 scenario in Iran, by the 2030s (January = 1; December = 12). The map was generated using ArcGIS v10.5 (www.esri.com).

**Figure 3:**
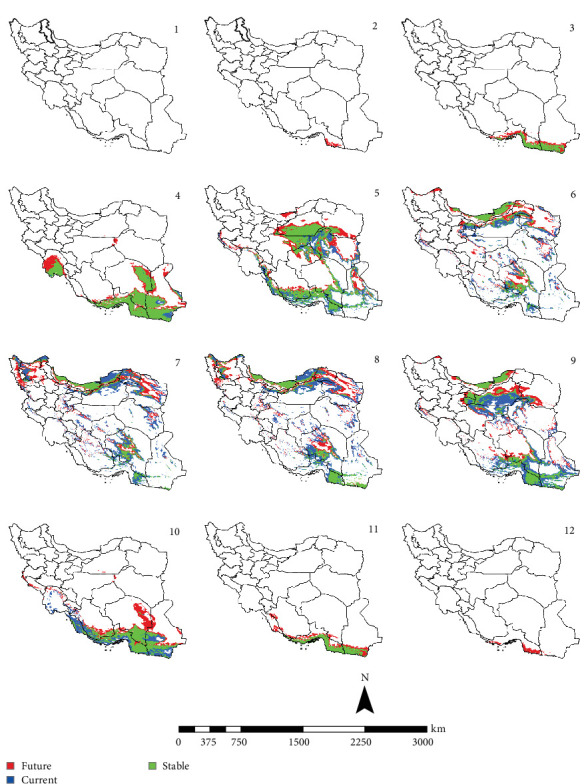
The projection of the activity of *Phlebotomus papatasi* under SSP5-8.5 scenario in Iran, by the 2030s (January = 1; December = 12). The map was generated using ArcGIS v10.5 (www.esri.com).

**Figure 4:**
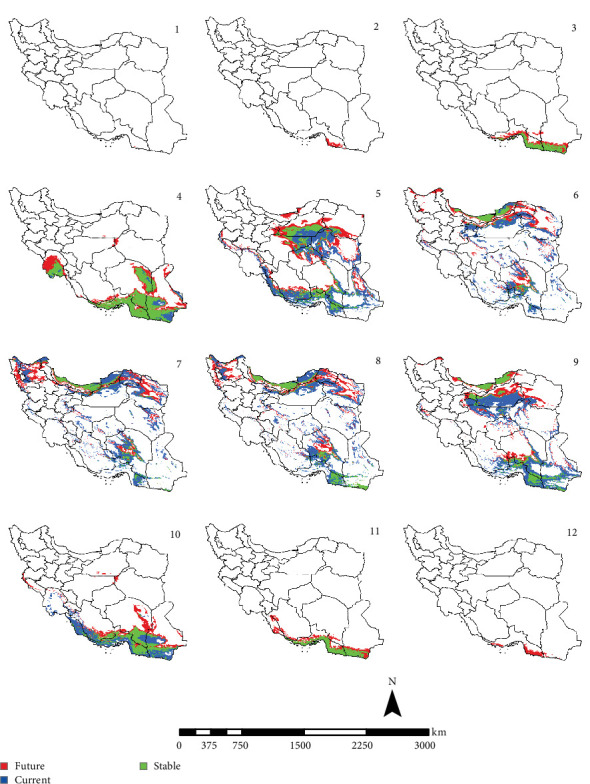
The projection of the activity of *Phlebotomus papatasi* under SSP1-2.6 scenario in Iran, by the 2050s (January = 1; December = 12). The map was generated using ArcGIS v10.5 (www.esri.com).

**Figure 5:**
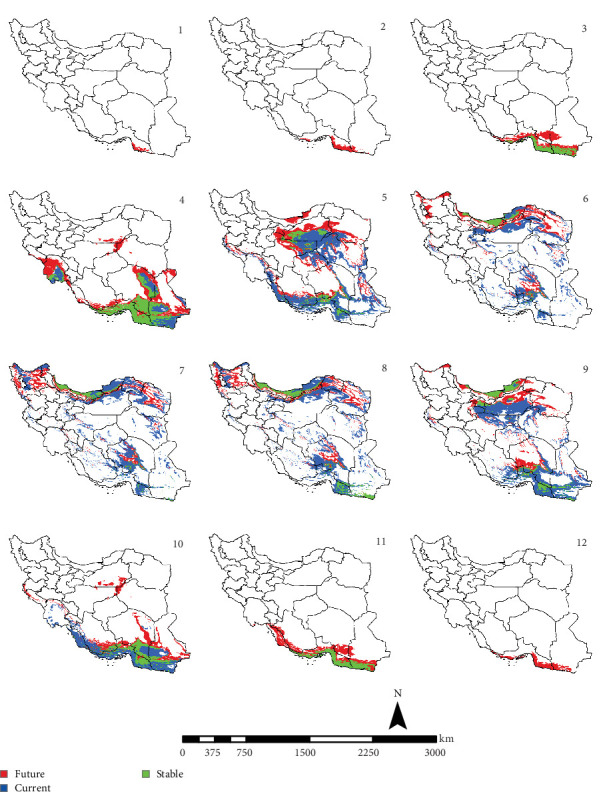
The projection of the activity of *Phlebotomus papatasi* under SSP5-8.5 scenario in Iran, by the 2050s (January = 1; December = 12). The map was generated using ArcGIS v10.5 (www.esri.com).

In the reference period, the period 1970–2000 which represents the current climate conditions, *Ph. papatasi* appeared from March to November. In March, *Ph. papatasi* was concentrated in the southern region, and from April to October, it was mainly concentrated in more regions of the country. In the southern and southeastern regions, including Sistan–Baluchestan and Hormozgan provinces, the active period now extends from March to November. In the reference period, the activity month of *Ph. papatasi* started in May and ends in September in Ilam, Esfahan, and Semnan provinces. On the other hand, its activity month started in the Khuzestan and Golestan provinces in April and June, respectively. Also, it ends in October and September in the two mentioned provinces, respectively (Figures 2–5).

In the period of the 2030s and under both scenarios (SSP126 and SSP585), the monthly activity of the species will start around February and end at the turn of December in Iran. In other words, compared to the current conditions, it starts its activity 1 month earlier and ends 1 month later (Figures 2 and 3). The longest period of activity will be in the Sistan–Baluchistan and Hormozgan provinces. This species will likely have monthly activity in Khuzestan, Semnan, and Golestan provinces until the 2050s, similar to the reference period. But in the east of Esfahan and south of Semnan Provinces, the length of the activity period will likely extend by 2 months, commencing in April and continuing through October. Also according to the SSP5-8.5-2050s' scenario, this vector can be active whole year in the Hormozgan Province. There will likely be a significant increase in the activity of this species in November in the Sistan–Baluchestan, Hormozgan, Bushehr, South of Kerman, and South of Fars provinces. In general, areas with higher latitudes are likely to be more affected by global warming and provide suitable temperature conditions for the activity of *Ph. papatasi* (Figures 4 and 5).

### 3.2. Projection of Temperature Changes Under Different Climate Change Scenarios in Iran

**Figure 6:**
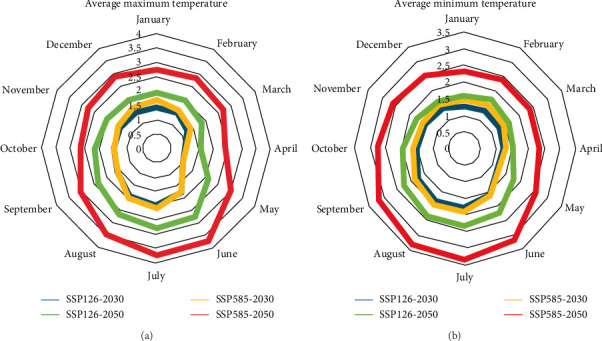
The difference between the monthly average (A) maximum and (B) minimum temperature (°C) of the 2030s and 2050s with the current conditions according to the MIROC6 climate model and under SSP1-2.6 and SSP5-8.5 climate scenarios in Iran.

According to both scenarios, an increasing trend of temperature can be expected in both periods of the 2030 and 2050s, and temperature changes are greater in the 2050s than in the 2030s. The maximum amount of changes is related to the summer (June to August). According to both scenarios, the average maximum temperature will increase more than the average minimum temperature by the 2050s. This situation is the opposite in 2030. So that the average minimum temperature will increase more than the average maximum temperature by 2030s (Figure 6).

### 3.3. Field Evaluation of *Ph. papatasi* Monthly Activity

The presence/absence results for *Ph. papatasi* showed that the model made a good prediction, and in all regions except Araghavanieh, this species was captured and identified.

### 3.4. The CL Situation in Iran and Selected Foci

**Figure 7:**
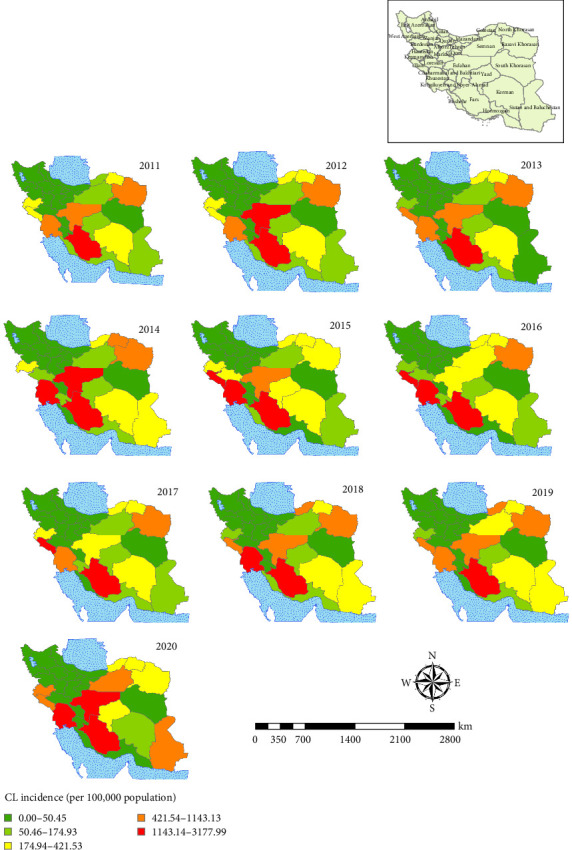
Annual incidence of cutaneous leishmaniasis in Iran by province, 2011–2020. The map was generated using ArcGIS v10.5 (www.esri.com).

**Figure 8:**
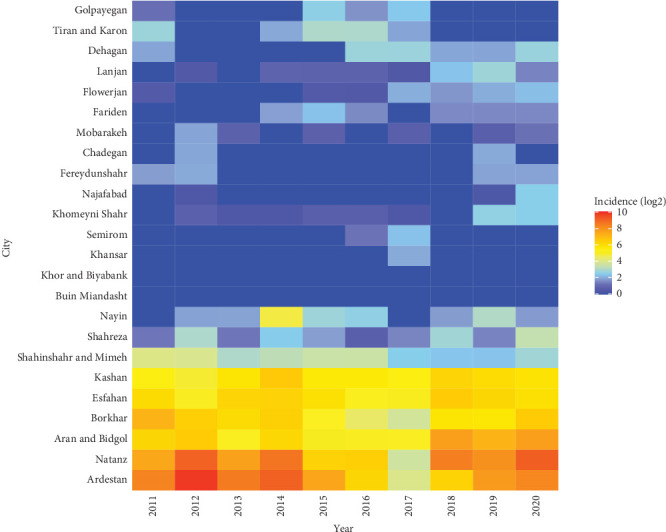
Annual incidence of cutaneous leishmaniasis in Esfahan Province, 2011–2020.

The spatial map of CL is shown at the province levels during 2011–2020 (Figure 7). The state of this disease in the country shows that provinces such as Fars, Esfahan, Khuzestan, and Ilam have the highest incidence of the disease in the last 10 years. In Esfahan Province, the highest cumulative incidence is related to the years 2011, 2013, and 2019, and in those years, the incidence of the disease was between 1100 and 3100 per 100,000 people (Figure 7). The highest incidence of the CL is related to Ardestan and Natanz (Figure 8). We considered Ardestan City in Esfahan Province as the selected foci to evaluate our predictions. The disease in Ardestan has been decreasing from 2016 to 2018, and then it has been increasing again (Figure 8).

### 3.5. Relationships Between ZCL With *Ph. papatasi* and Environmental Variables

**Figure 9:**
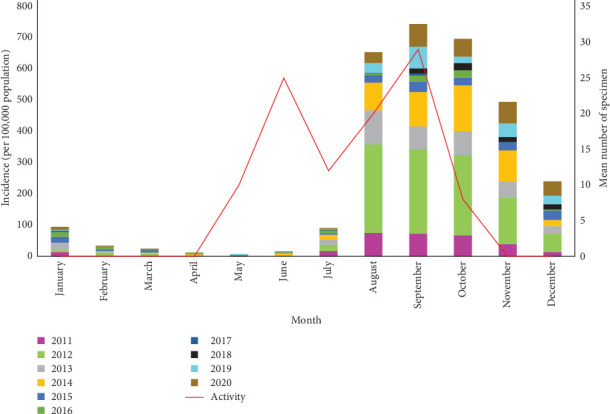
Monthly activity of *Phlebotomus papatasi* (secondary axis) and the monthly incidence of zoonotic cutaneous leishmaniasis (ZCL) (primary axis) in Ardestan, 2011–2020.

**Figure 10:**
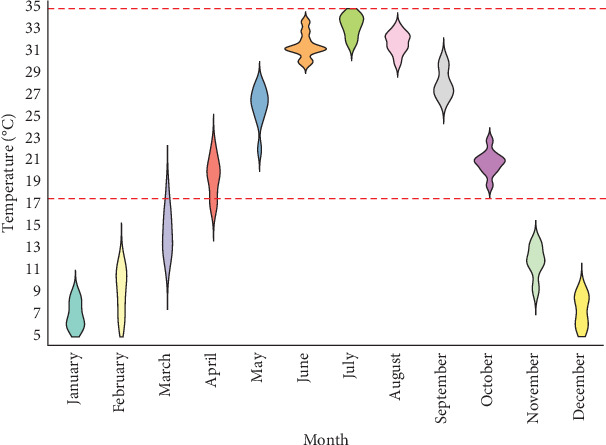
Monthly average temperature in selected foci of zoonotic cutaneous leishmaniasis (ZCL) in Ardestan, 2011–2020. The horizontal black lines indicate lower (17.63°C) and upper (35°C) threshold temperatures for the growth of the *Phlebotomus papatasi* species.

The monthly activity of *Ph. papatasi* has two peaks. The first and second peaks occur in June and September, respectively (Figure 9). The temperature range of 17.63°–35° which is optimal for the growth of the *Ph. papatasi* is specified in Figure 10. From 2011 to 2020 in Ardestan, the average monthly temperature increased from March and April and reached its peak in July and then decreased. The average maximum and minimum temperature in this place are 35.24 and 4.65, respectively. In general, the average temperature in this city is 20.13 (Figure 10).

The ZCL incidence increases 2–4 months after the peak of the vector activity. Therefore, the peak of the ZCL incidence shows that the disease mostly appears in the autumn. The second peak of the disease occurs in early winter. The highest incidence of the disease in Ardestan is in autumn, and the cumulative graph of the incidence of ZCL in this city in September is 700 per 100,000 population (Figure 9).

Statistically, as shown in column B in Table 1, the results of Poisson regression analysis show that the logarithm of the incidence increases by 1.36 in Ardestan with the increase of every single monthly average temperature. In other words, as shown in column Exp (B) in Table 1, with an increase of every single degree in the monthly average temperature, the average ZCL incidence is multiplied by 3.911, which indicates the increase in the incidence of the ZCL in Ardestan. In general, under the existing conditions, the incidence of the ZCL increases by 0.364 per month. Given that sig. (*p* ≤ 0.001) and considering that the confidence interval for Exp (B) does not include any of zero, there was a significant correlation between the incidences of ZCL and with monthly average temperature in Ardestan. Increasing the maximum and minimum temperature has a reducing effect on the disease. With the increase of each degree of maximum and minimum temperature, the incidence of the disease decreases by 0.885 and 0.257 (*p* ≤ 0.001) (Table 1).

## 4. Discussion

Iran has a diverse climate, with cool and subhumid conditions in the west, cold and temperate semiarid climate in the northwest, warm and semiarid conditions in the southwest, and warm and hyperarid conditions in the southeast and central regions [35]. This provides an excellent opportunity to study the impact of climate change on species distribution and disease transmission [36]. Iran is among the countries projected to be significantly affected by global warming in the future [23]. This study explored the dynamics of *Ph. papatasi*, a key vector CL, and its relationship with temperature changes in Iran. Our study projects that *Ph. papatasi* will have an extended activity period in the coming decades, particularly in regions where temperatures are expected to increase. At temperatures above 35°C, the activity of *Ph. papatasi* slows down [21], but lower temperatures may prevent its full development [37]. These combined effects suggest that climate change will shift the timing and length of activity [38, 39], especially in regions such as Esfahan, where we project a longer activity. In other words, our results indicate that with future temperature changes, *Ph. papatasi* will experience an extended period of activity in many regions of the country. The month-length activity, which currently spans March to November, may increase by 1 to 2 months in some regions. For instance, in central provinces like Esfahan, peak activity might shift earlier to April, with a secondary peak extending into September and October.

The data are supported by other studies in countries in the Mediterranean basin. Studies by Trájer [25] and Bounoua et al. [26] confirm that increased temperatures in regions like North Africa, Southern Europe, and the Middle East lead to an expansion of the active period for sandfly populations. These findings support the claim that the month-length activity of *Ph. papatasi* will continue to increase, making more months of the year conducive to the survival and reproduction of the vector. The increase in the activity period of *Ph. papatasi* has been documented in other regions with warming temperatures. For example, research conducted in the Mediterranean basin predicts similar patterns. These studies have noted an expansion of the vector's activity season due to temperature changes, driven by climate change, and phlebotomine sand flies are prospected to invade extra-Mediterranean regions, especially western and central Europe [40].

Therefore, the impact of temperature changes on *Ph. papatasi* activity varies across regions [8, 25, 41]. In northern and high-latitude parts of Iran, the activity period is generally shorter, whereas southern regions such as Sistan–Baluchestan and Hormozgan will likely see a longer activity, extending from March to November. This variability reflects the diversity of Iran's climate and topography. Our findings align with other regional studies, such as those in Golestan, where *Ph. papatasi* is active from June to October, peaking in August [22], and Jask, where activity lasts until December [27]. However, our projections indicate an earlier start in central regions like Esfahan, potentially shifting the peak activity to April due to rising temperatures. In central provinces, warmer summers may alter the activity patterns of *Ph. papatasi* or lead to a summer diapause. For instance, in Esfahan, peak activity shifts from June, July, and August to April, while a second peak occurs from September to October.

Notably, in southern Semnan, the peak activity might shift from September to October due to future climate conditions. In general, most regions will see activity from June to October, with significant variations based on local climate conditions. Although there are desert conditions in the south of Semnan Province, our study highlighted that the environmental conditions in terms of the investigated factors (average minimum and maximum monthly temperature) will be suitable for the activity of *Ph. papatasi*. However, this does not guarantee the presence of the vector, as other ecological factors such as land use, vegetation cover, and human interventions may also influence its distribution. Furthermore, although climatic models predict favorable temperature conditions for its activity, further field studies are necessary to confirm these projections and assess other environmental variables that could affect the species' survival and proliferation. Such findings could have significant implications for the management and control of VBDs like CL in these regions, particularly as the vector's activity extends with shifting climatic patterns. We evaluated the modeling by sampling six randomly selected districts in Esfahan Province in July when temperatures are ideal for *Ph. papatasi*. Our predictions were mostly accurate, though sampling bias could influence model performance. Previous field evaluations support the reliability of these models for future projections [24, 42, 43]. Despite expecting to find the species in Araghavanieh, no specimens were captured, likely due to land use changes. Interestingly, our study found discrepancies between laboratory predictions and field observations. In northern Iran, *Ph. papatasi* is active all year long in laboratory conditions [44], but our projections show only 4 months of activity outdoors. This difference likely arises because laboratory conditions do not fully capture the environmental complexity, such as indoor activity, where 85% of *Ph. papatasi* specimens were caught.

It is not surprising that any change in the temporal and spatial distribution will lead to a change in the pattern of ZCL in the country [45]. Temperature plays a crucial role in the physiology and metabolism of *Phlebotomus* species. Simply stated, temperature impacts directly the developmental periods and survival of sand flies [21, 46], and the extended activity of *Ph. papatasi* under future temperature changes has significant implications for the spread of ZCL [24, 26, 47–49]. Furthermore, most temperature changes are related to higher latitudes [50], and the north and northwest parts of the country will be suitable for *Ph. papatasi* activity in the future [24]. There is evidence from Iran that shows CL is moving to higher latitudes [51], which may be due to global climate change [52]. Consistent with this fact, Maroli et al. [53] showed that leishmaniasis has spread in a northeward direction and toward higher latitudes in Italy in the last decades.

This spatial expansion and extended activity period could increase the risk of CL transmission in these areas, and previous studies have shown that the incidence of CL is closely related to vector abundance, with peaks in disease occurring 2–4 months after the peak of vector activity [15]. Our projections suggest that as the vector's activity extends, particularly in high-risk provinces like Esfahan and Khuzestan, the transmission season for CL could also lengthen, leading to more cases in previously unaffected areas. Confirmed CL cases are typically diagnosed at the genus level; however, for research purposes, investigators may identify cases down to the species level when needed [4]. Epidemiological evidence in selected foci, Ardestan, indicates that CL cases are exclusively attributed to *L. major*, with *Ph. papatasi* identified as the primary vector responsible for ZCL transmission in the region [54]. Given this well-established association, we can assert with confidence that the CL cases included in our study are indeed cases of ZCL. Consequently, since our analyses were conducted in Ardestan, where *Ph. papatasi* is the dominant vector and *L. major* is the sole causative agent, we are certain that the findings presented in this study are specific to ZCL. This provides a clear epidemiological basis for linking our results to the dynamics of ZCL transmission in the region. Considering that activity of ZCL vectors in Iran is mostly related to May to September [8, 55] and the ZCL incidence will increase 2–4 months after the peak of the vectors' activity [15], we expected that ZCL cases appear in Ardestan mostly in the autumn and/or winter. Over the past 10 years, the data analysis in Ardestan has consistently revealed a similar trend, aligning with our findings. The monthly pattern of CL has been shown in some other studies [10–12, 36, 56].

In the context of SSPs, particularly the SSP5-8.5 scenario, which represents a high-emission, fossil-fuel-dependent future, the data project a substantial rise in the monthly activity of *Ph. papatasi* by 2050. This predicted increase is linked to warmer temperatures, which create favorable conditions for vector survival and reproduction, thereby expanding their active periods throughout the year. This trend mirrors previous research, such as [25], which demonstrated that climate change is directly responsible for shifts in vector behavior and the spread of diseases like ZCL. Interestingly, the effect of climate change is not uniform across regions. While some provinces like Ilam, Khuzestan, and Bushehr may experience a decline in the probability of *Ph. papatasi* presence, potentially due to extreme heat or other climatic factors making these regions less favorable for the vector, other areas are expected to emerge as new hotspots. These new hotspots include provinces such as Kurdistan, Azerbaijan–Gharbi, Alborz, and northern Semnan, where the climate may become more suitable for *Ph. papatasi* activity. This shift underscores the dynamic nature of VBD distribution, heavily influenced by changing environmental conditions. In addition to the findings related to the relationship between the temperature and the activity of *Ph. papatasi*.

Although various studies indicate that relative humidity can also be linked to the disease pattern differently across various regions [57, 58] and the influence of relative humidity and other factors such as precipitation on the activity of this species could provide further insights, unfortunately, there is limited specific documentation regarding the precise ranges of precipitation and relative humidity on order to predict their effects on *Ph. papatasi'*s activity. In the future, studies should aim to investigate the role of these additional weather factors in more detail.

## 5. Conclusion

This study underscores the profound impact that climate change is projected to have on the distribution and activity patterns of *Ph. papatasi*, a key vector for ZCL in Iran. With the country's diverse climate ranging from cool and subhumid regions in the west to hyperarid conditions in the southeast, Iran provides an ideal setting for studying the relationship between temperature variations and VBDs. Our findings project an extended activity period for *Ph. papatasi* in many regions, particularly under the SSP5-8.5 climate scenario, which anticipates substantial increases in temperatures by 2050. While temperature is a dominant factor in shaping *Ph. papatasi*'s activity, its influence is not uniform across Iran. This regional variability highlights the need for localized public health strategies to manage the risks of ZCL transmission as climate conditions evolve.

Moreover, the correlation between vector abundance and ZCL incidence, with disease peaks occurring 2–4 months after the peak in *Ph. papatasi* activity, suggests that the extended activity period will likely lead to longer transmission seasons in many regions. However, additional factors such as land use, human interventions, and vegetation cover also play critical roles in vector distribution, emphasizing the need for further field studies to refine these projections and account for ecological complexity. While temperature remains a critical determinant of *Ph. papatasi* activity, future research should explore the role of other environmental factors such as precipitation and relative humidity, which could also influence vector dynamics and disease transmission patterns. Understanding the combined effects of these variables will be essential for developing comprehensive strategies to mitigate the impact of climate change on ZCL transmission in Iran and other affected regions.

## Figures and Tables

**Table 1:** Regression analysis results between the incidence of zoonotic cutaneous leishmaniasis (ZCL) with abundance of *Phlebotomus papatasi* and environmental variables in Ardestan, 2011–2020.

			Hypothesis test				99% Wald confidence interval for exp (B)	
Variables	B	Std. error	Wald chi-square	df	Sig.	Exp (B)	Lower	Upper
Monthly average temperature	1.36	0.043	993.91	1	*p* < 0.001	3.911	3.593	4.257
Monthly average maximum temperature	−0.885	0.034	643.25	1	*p* < 0.001	0.413	0.385	0.442
Monthly average minimum temperature	−0.257	0.044	33.456	1	*p* < 0.001	0.773	0.709	0.844
Abundance of *Ph. papatasi*	0.001	4.4734E-5	318.348	1	*p* < 0.001	1.001	1.001	1.001
Month	0.364	0.015	527.822	1	*p* < 0.001	1.439	1.395	1.484

## Data Availability

The datasets used and analyzed during the current study are available from the corresponding author upon reasonable request.
